# Zeb1-induced metabolic reprogramming of glycolysis is essential for macrophage polarization in breast cancer

**DOI:** 10.1038/s41419-022-04632-z

**Published:** 2022-03-04

**Authors:** Huimin Jiang, Huimin Wei, Hang Wang, Zhaoyang Wang, Jianjun Li, Yang Ou, Xuechun Xiao, Wenhao Wang, Antao Chang, Wei Sun, Li Zhao, Shuang Yang

**Affiliations:** 1 Tianjin Key Laboratory of Tumor Microenvironment and Neurovascular Regulation, Medical College of Nankai University, Tianjin, 300071 China; 2 Laboratory of Brain Disorders, Ministry of Science and Technology, Collaborative Innovation Center for Brain Disorders, Beijing Institute of Brain Disorders, Beijing Advanced Innovation Center for Big Data-based Precision Medicine, Capital Medical University, Beijing, 100069 China; 3 Beijing Advanced Innovation Center for Big Data-Based Precision Medicine, School of Engineering Medicine, Beihang University, Beijing, 100191 China; 4 Department of Biochemistry and Molecular Biology, School of Basic Medical Sciences, National Clinical Research Center for Cancer, Key Laboratory of Cancer Prevention and Therapy, Tianjin’s Clinical Research Center for Cancer, Tianjin Medical University Cancer Institute and Hospital, Tianjin Medical University, Tianjin, 300070 China

**Keywords:** Breast cancer, Cancer metabolism, Tumour immunology

## Abstract

Aerobic glycolysis (the Warburg effect) has been demonstrated to facilitate tumor progression by producing lactate, which has important roles as a proinflammatory and immunosuppressive mediator. However, how aerobic glycolysis is directly regulated is largely unknown. Here, we show that ectopic Zeb1 directly increases the transcriptional expression of HK2, PFKP, and PKM2, which are glycolytic rate-determining enzymes, thus promoting the Warburg effect and breast cancer proliferation, migration, and chemoresistance in vitro and in vivo. In addition, Zeb1 exerts its biological effects to induce glycolytic activity in response to hypoxia via the PI3K/Akt/HIF-1α signaling axis, which contributes to fostering an immunosuppressive tumor microenvironment (TME). Mechanistically, breast cancer cells with ectopic Zeb1 expression produce lactate in the acidic tumor milieu to induce the alternatively activated (M2) macrophage phenotype through stimulation of the PKA/CREB signaling pathway. Clinically, the expression of Zeb1 is positively correlated with dysregulation of aerobic glycolysis, accumulation of M2-like tumor-associated macrophages (TAMs) and a poor prognosis in breast cancer patients. In conclusion, these findings identify a Zeb1-dependent mechanism as a driver of breast cancer progression that acts by stimulating tumor–macrophage interplay, which could be a viable therapeutic target for the treatment of advanced human cancers.

## Introduction

The reprogramming of energy metabolism characterized by high glycolysis even in the presence of abundant oxygen is recognized as a hallmark of cancer [1]. During this process, cancer cells exhibit increased glucose uptake and convert pyruvate into lactate under aerobic conditions, resulting in a state referred to as aerobic glycolysis or the Warburg effect [2, 3]. This metabolic adaptation is critical for cancer cell proliferation, invasion, metastasis and particularly the response to therapeutic intervention [4–11]. Therefore, cancer cells express metabolic enzymes and transporters involved in glycolysis at higher levels than normal cells [12]. The enhanced glycolysis in cancer cells leads to lactate accumulation within the tumor microenvironment (TME), which is correlated with malignant progression and a poor prognosis in various human cancers, including breast cancer [13, 14]. For example, increased lactate levels are instrumental in the promotion of tumor angiogenesis through a hypoxia/HIF-1α-dependent mechanism [15, 16]. In addition, the glycolytic switch in cancer cells also limits the development of an effective antitumor immune response [10, 17, 18].

Macrophages are prominent immune cells in the TME that exert potent effects on tumorigenesis [19, 20]. Depending on signals present in the microenvironment, macrophages are polarized into one of two distinct phenotypes: the classically activated (M1) or alternatively activated (M2) phenotype [21, 22]. Increasing evidence has demonstrated that tumor-associated macrophages (TAMs), which are characterized by typical M2-like macrophage properties, are critical players in the crosstalk between cancer cells and their microenvironment [23, 24]. TAMs can secrete cytokines (e.g., IL-6, IL-10, and TGF-β) to suppress the anticancer immune response and thus foster cancer progression [24–26]. Moreover, microenvironmental cues, such as chemokines (e.g., CSF-1 and CCL2), alterations to the extracellular matrix and even hypoxic conditions, contribute to the recruitment and immunosuppressive functions of TAMs [27, 28]. Recent studies have also shown that aerobic glycolysis in cancer cells leads to the accumulation of extracellular lactate, which in turn educates macrophages to become functional TAMs within the TME [29–31]. Nevertheless, the underlying mechanisms involved in the communication between cancer cell-derived lactate and TAMs are poorly defined.

Zinc finger E-box binding homeobox 1 (Zeb1) is a transcription factor that influences developmental and homeostatic cell fate decisions in a broad range of tissues [32–35]. Mechanistically, Zeb1 can directly activate or repress gene expression by binding to the regulatory regions of target genes [36–38]. Recent evidence has suggested that aberrant expression of Zeb1, which is mostly found at the invasive front of carcinomas, promotes the malignant progression of breast cancer and other cancer types [39–46]. Zeb1 endows cancer cells with pro-invasive and stem-like phenotypes and determines a relatively poor clinical prognosis in human cancer patients [37, 38, 42, 46–50]. However, whether Zeb1 regulates the aberrant metabolism of cancer glycolysis is unclear.

In this study, we identified Zeb1 as a key regulator of tumorigenesis-associated metabolic reprogramming by directly targeting glycolytic enzymes in breast cancer. Moreover, the accumulation of lactate derived from cancer cells with ectopic Zeb1 was found to contribute to the M2-like polarization of TAMs in the TME and thus promote the malignant progression of breast cancer in vitro and in vivo. Together, our data suggest the presence of important interplay between the endogenous metabolic dysregulation and development of an immunosuppressive TME through a Zeb1-dependent mechanism, highlighting the possibility of identifying new targets and therapeutic strategies for cancer treatment.

## Materials and methods

### Generation of conditional *Zeb1*^−/−^ mice

To generate a conditional Zeb1 knockout allele (Zeb1^*fl/fl*^), exon 6 was flanked by loxP sites to remove sequences coding for large parts of the protein and to induce a premature translational stop codon. MMTV-PyMT mice (a mouse model of spontaneous breast cancer) were crossed with Zeb1^*fl/fl*^ mice to generate *Zeb1*^*fl*/*fl*^PyMT^+/−^ mice (PyMT), which were then crossed with MMTV-Cre mice to generate *Zeb1*^−/−^PyMT^+/−^Cre^+/−^mice (PyMT;*Zeb1*^*cKO*^). PyMT and PyMT;*Zeb1*^*cKO*^ offspring were palpated weekly to monitor for tumor initiation. Mice were handled in accordance with protocols approved by the Animal Care and Use Committees of the Medical College of Nankai University.

### Genotyping

PCR was performed using DNA from tail biopsies. All mice were genotyped to evaluate the *MMTV-PyMT*, *MMTV-Cre*, and *Zeb1* genes. The PCR primers for genotyping are listed in Supplementary Information.

### Primary cell lines

Tumors from PyMT and PyMT;*Zeb1*^*cKO*^ mice were minced with a razor blade, rinsed three times with PBS and digested with collagenase I (Sigma) at 37 °C with agitation for 30 min in DMEM supplemented with 2% fetal bovine serum (FBS) to prepare single-cell suspension. EpCAM^+^ cells as tumor epithelial cells were then sorted by a FACS Aria instrument (BD). To establish tumor cell lines, 1 × 10^7^ dissociated and filtered tumor cells were plated in a 10-cm dish in DMEM supplemented with 10% fetal bovine serum, 1% sodium pyruvate and 1% Pen/Strep. The next day, dead cells were removed with a medium change, and the attached cells were used for subsequent experiments. Tumor cell lines were all derived from high-grade carcinomas in 13- to 16-week-old females.

### Cell culture

MDA-MB-231 and THP-1 cells were cultured with RPMI-1640 medium supplemented with 10% fetal bovine serum (FBS) at 37 °C in 5% CO_2_. SUM-159 cells were cultured with high-glucose Dulbecco’s modified Eagle’s medium (DMEM) supplemented with 10% FBS at 37 °C in 5% CO_2_. HEK293T cells were cultured with DMEM supplemented with 10% FBS, 1% sodium pyruvate, 1% NEAA and 2% glutamine at 37 °C in 5% CO_2_. Authenticated cell lines were purchased from ATCC (American Type Culture Collection) and was not contaminated by mycoplasma_._ For M2-like TAMs generation, THP-1 cells were stimulated with 200 ng/mL phorbol 12-myristate 13-acetate (PMA, Sigma) for 24 h and polarized into macrophages (THP-1 MΦ), followed by further incubation with conditioned medium or lactate for another 24 h [51, 52].

### Lentiviral knockdown and expression system

Zeb1-specific shRNA was annealed and subcloned into the pLV-H1-EF1α-puro vector (Biosettia). The Zeb1 cDNA sequence was subcloned into the pLV-EF1-MCS-IRES-Bsd vector (Biosettia). HEK293T cells were then cotransfected with lentiviral vectors and packaging plasmids using Lipofectamine 2000 reagent (Invitrogen) to generate lentiviral particles. Viral supernatants were collected 48 h after transfection, centrifuged at 75,000 × *g* for 90 min, resuspended and filtered through 0.45-μm filters (Millipore).

### RNA extraction and quantitative RT-PCR

Total RNA was extracted from breast cancer cells and freshly sorted primary tumor cells using TRIzol (Life Technologies). cDNA was synthesized using M-MLV Reverse Transcriptase (Takara). The specific Zeb1 products were amplified by quantitative PCR using the TransStart Green Q-PCR SuperMix Kit (TransGen). β-actin was used as a normalization control. The primer sequences are listed in Supplementary Table 1.

### RNA-sequencing and gene-set enrichment analysis (GSEA)

RNA sequencing was performed following the pipeline of BGI-tech (BGI). Briefly, total RNA was isolated using Trizol reagent. Total RNA was processed by mRNA enrichment: The mRNA with polyA tail was enriched by magnetic beads with OligodT. The RNA obtained was segmented by interrupting buffer, and the random N6 primers were reversely transcribed, and then the cDNA two-strand was synthesized to form double-stranded DNA. The synthetic double-stranded DNA are flattened and phosphorylated at the 5’-end and formed a sticky end protruding an “A” at the 3′-end, followed by a bubbling-like connector protruding “T” at the 3′-end. The linked products were amplified by PCR using specific primers. Primary sequencing data were produced by MGISEQ-2000. After quality control, raw reads were filtered into clean reads and then processed by the tophat and cufflinks algorithms. GSEA was performed by R (ClusterProfiler package) with selected parameters: nPerm = 1000, minGSSize = 10, maxGSSize = 500, *P*-valueCutoff = 0.05, and pAdjustMethod = “BH” [53]. The RNA-Seq data are available at the NCBI Gene Expression Omnibus under accession number GSE189779.

### Immunoblotting assay

Preparation of total cell extracts and immunoblotting with appropriate antibodies were performed as previously described [54]. The appropriate antibodies were used as described in Supplementary Table 2. Labeled proteins were visualized with an ECL chemiluminescence kit (Millipore).

### Chromatin immunoprecipitation (ChIP)

ChIP assays were performed using an EZ-ChIP kit (Millipore) according to the manufacturer’s instructions. Briefly, cells were crosslinked with 1% formaldehyde for 10 min at room temperature, and the formaldehyde was then inactivated by addition of 125 mM glycine. Chromatin extracts containing DNA fragments were immunoprecipitated using specific antibodies. The ChIP-enriched DNA was then uncrosslinked and subjected to quantitative PCR. Data were analyzed using the SuperArray ChIP-qPCR Data Analysis Template (Qiagen/SABiosciences) [55, 56]. The primers and antibodies used in these experiments are shown in Supplementary Tables 1 and 2.

### HK, PFK, and PK activity assays

The enzyme activities of HK, PFK, and PK in cancer cells were measured using a hexokinase colorimetric assay kit (BioVision), phosphofructokinase (PFK) activity colorimetric assay kit (BioVision), and pyruvate kinase activity colorimetric/fluorometric assay kit (BioVision), respectively, following the manufacturer’s protocols.

### Cellular respiration (OCR) and extracellular acidification (ECAR) assays

OCR and ECAR measurements were performed by using the Seahorse XF24 Extracellular Flux Analyzer (Seahorse, USA). After trypsinization, cancer cells were seeded in Seahorse XF24 cell plates (2.5 × 10^4^/well) in a humidified 37 °C, 5% CO_2_ incubator for 24–48 h. The cell plates were then placed in a 37 °C, 0% CO_2_ incubator for 60 min prior to the start of an assay. A glycolytic stress test kit (Seahorse) and mitochondrial stress test kit (Seahorse) were used to detect glycolytic flux and mitochondrial metabolic flux, respectively, following the manufacturer’s protocols.

### Glucose uptake assay

Cells were seeded in six-well plates (2 × 10^5^/well) and incubated at 37 °C for 24 h. Prior to an assay, cells were deprived of glucose for 2–3 h by exchanging the growth medium with glucose-free medium. The cell medium was then changed to fresh medium with or without fluorescent 2-NBDG (Thermo). The cells were incubated at 37 °C for an additional 45 min, washed with PBS and subsequently digested into single cells for analysis.

### Pyruvate, lactate, and ATP measurements

The production of pyruvate, lactate, and ATP by cancer cells was measured using a pyruvate assay kit (BioVision), lactate colorimetric assay kit (BioVision), and ATP colorimetric/fluorometric assay kit (BioVision), respectively, following the manufacturer’s protocols.

### Luciferase assay

Cells were transfected with the wild-type or mutant human HK2, PFKP, or PKM2 promoters, followed by transfection with a Zeb1 expression vector in 24-well plates. Lysates were prepared 48 h after transfection, and luciferase activity was measured using the Dual-Luciferase Reporter Assay System (Promega) according to the manufacturer’s protocols. Luciferase activity was normalized to the values for Renilla luciferase.

### Cell viability assay

A total of 3 × 10^3^ cells/well were seeded in 96-well plates and cultured for 24 h. The cells were then treated with oxamic acid (OXM) in the presence or absence of EPI/ETOP. At the indicated time points, cell viability was measured using a cell counting kit-8 (CCK-8, Dojindo) assay in accordance with the manufacturer’s instructions. The absorbance values were measured at 450 nm. Six parallel wells were assigned for each group.

### Wound-healing assay

Cells were seeded in a six-well plate and allowed to grow to confluence. The cell monolayer was subsequently scratched with a 10-μL pipette tip to create a narrow wound-like gap. The cells were then washed twice with PBS, fed conditioned medium containing 1% FBS, and treated with OXM. Images were acquired under a light microscope (Olympus), and the migration distance was quantified using ImageJ software.

### Transwell assay

Cells were placed in the top chambers in the presence or absence of OXM and allowed to migrate. After 20 h, the non-migrated cells were removed with a cotton swab, and the migrated cells were fixed with 20% methanol and stained with 0.5% crystal violet. The cells were counted and imaged under a light microscope (Olympus).

### Tumor xenograft experiments

In each animal experiment, mice were randomly allocated to each group. All of the experimental procedures involving animals were performed in accordance with a protocol approved by the Ethics Committee for Animal Use at the Medical College of Nankai University. Six-week-old female BALB/c nude mice were used. Cells were injected into the mouse mammary fat pads. Tumors were allowed to develop for 10 days. The mice were then intraperitoneally injected with 5 mg/kg EPI and/or 20 mg/kg OXM (once every 2 days) for another 2 weeks. The mice were then sacrificed, and the volume and weight of tumors were analyzed.

### Tissue microarray and immunohistochemistry (IHC) scoring

A total of 128 samples of human breast invasive ductal carcinoma were obtained from Shanghai Outdo Biotech Co., Ltd., China. This project was approved by the Institute Research Ethics Committee of the above institution. The samples were stained with the appropriate antibodies (Supplementary Table 2) using the Envision Kit (Dako) following the manufacturer’s protocol. The immunostaining was independently evaluated by 2 pathologists. The IHC score was calculated as previously described [57].

### Statistical analysis

Statistical analyses were performed using GraphPad Prism 7.0 and SPSS 13.0 software. All the data are presented as the mean ± SD and represent at least three independent experiments. Spearman’s rank correlation test was used to analyze correlations of gene expression in tissue samples. One-way analysis of variance (ANOVA) was used to compare means among treatment groups. Where appropriate, Student’s *t*-test for unpaired observations was applied. A *P*-value < 0.05 was considered significant. The *r*-value test was used to evaluate the correlation analysis.

## Results

### Zeb1 is a key regulator of glycolytic gene expression

**Fig. 1:**
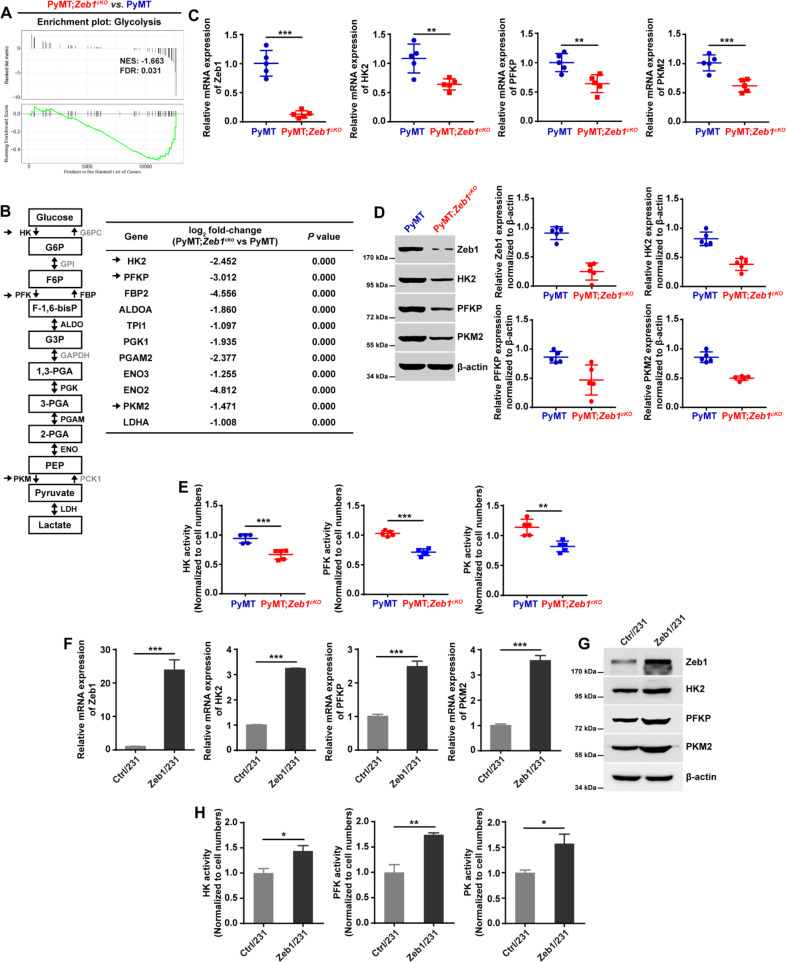
Zeb1 is a key regulator of glycolytic gene expression. **A** GSEA of transcriptome data from PyMT;*Zeb1*^*cKO*^ vs PyMT cells revealing enrichment of a gene signature associated with reduced glycolysis in PyMT;*Zeb1*^*cKO*^ breast cancer cells. NES, normalized enrichment score; FDR, false discovery rate. **B** Differential expression of glycolytic genes identified by RNA-sequencing. **C**, **D** The relative (**C**) mRNA and (**D**) protein levels of Zeb1, HK2, PFKP, and PKM2 in PyMT and PyMT;*Zeb1*^*cKO*^ cells (*n* = 5). **E** The enzyme activities of HK, PFK, and PKM in PyMT and PyMT;*Zeb1*^*cKO*^ cells (*n* = 5). **F**, **G** The relative (**F**) mRNA and (**G**) protein levels of HK2, PFKP, and PKM2 in empty vector-expressing (Ctrl/231) and Zeb1-expressing (Zeb1/231) MDA-MB-231 cells. **H** The enzyme activities of HK, PFK, and PKM in Ctrl/231 and Zeb1/231 cells. Dots depict individual samples in (**C**–**E**). Data are representative of five (**C**–**E**) or three (**F**–**H**) independent experiments and presented as mean ± SEM. *P < 0.05, **P < 0.01, ***P < 0.001 vs the respective control by an unpaired Student’s *t*-test.

To determine the role of Zeb1 in breast cancer development, we crossed the floxed *Zeb1* allele homozygously into PyMT mice to generate PyMT;*Zeb1*^*cKO*^ (*MMTV-Cre*^*+/−*^*;PyMT*^*+/−*^*;Zeb1*^*−/−*^) mice as previously described [50]. The EpCAM^+^ breast cancer epithelial cells were respectively isolated from PyMT and PyMT;*Zeb1*^*cKO*^ mice to perform RNA sequencing. The results of GSEA demonstrated that the loss of *Zeb1* expression in PyMT;*Zeb1*^*cKO*^ tumor cells led to downregulation of genes associated with the glycolysis signature compared with the enrichment observed in PyMT tumor cells (Fig. 1A). A total of 11 glycolysis-related genes were identified to be differentially expressed (Fig. 1B), including the genes encoding HK2, PFKP and PKM2, which are glycolytic rate-determining enzymes [5, 6]. Quantitative PCR (Fig. 1C), immunoblotting (Fig. 1D), and a colorimetric assay (Fig. 1E) further confirmed the decreased expression and reduced enzyme activity of HK2, PFKP, and PKM2 in PyMT;*Zeb1*^*cKO*^ tumor cells compared to PyMT cells. We also performed Zeb1 gain- and loss-of-function assays in MDA-MB-231 (Fig. 1F–H, Fig. S1) and SUM-159 (Fig. S2) human breast cancer cells. The results revealed that depletion of Zeb1 inhibited the expression and activity of HK2, PFKP and PKM2; however, ectopic Zeb1 expression had the opposite effect to enhance the glycolytic phenotypes in breast cancer cells. These observations identified Zeb1 as a key regulator of glycolytic gene expression.

### Zeb1 induces aerobic glycolysis

**Fig. 2:**
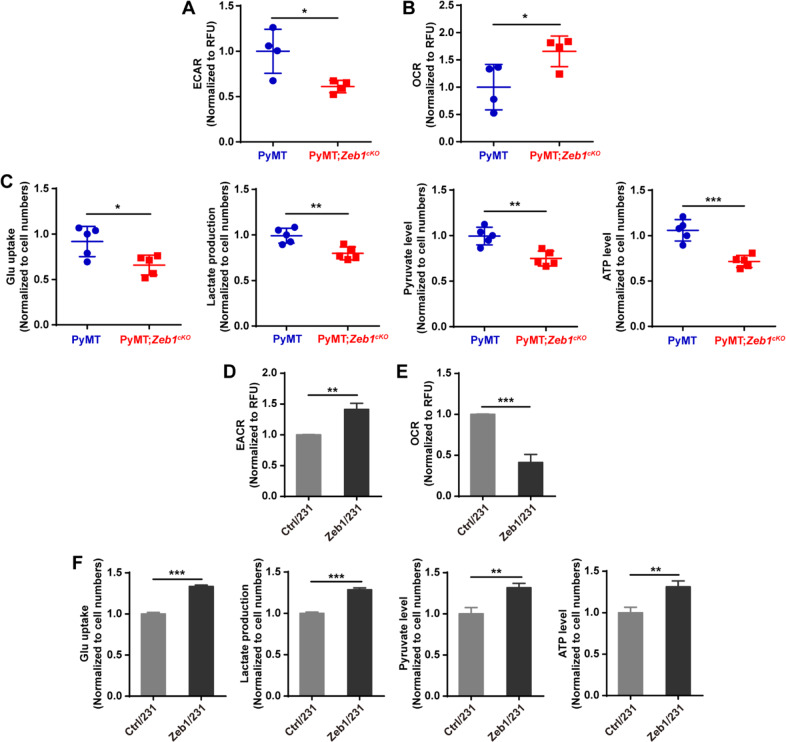
Ectopic Zeb1 induces aerobic glycolysis. **A**, **B** The alternations in (**A**) ECAR and (**B**) OCR in PyMT and PyMT;*Zeb1*^*cKO*^ cells (*n* = 5). **C** The alternations in glucose uptake, pyruvate level, lactate production, and ATP level in PyMT and PyMT;*Zeb1*^*cKO*^ cells (*n* = 5). **D**, **E** The alternations in (**D**) ECAR and (**E**) OCR in Ctrl/231 and Zeb1/231 cells. **F** The alternations in glucose uptake, pyruvate level, lactate production, and ATP level in Ctrl/231 and Zeb1/231 cells. Dots depict individual samples in (**A**–**C**). Data are representative of five (**A**–**C**) or three (**D**–**F**) independent experiments and presented as mean ± SEM. *P < 0.05, **P < 0.01, ***P < 0.001 vs the respective control by an unpaired Student’s *t*-test.

Next, we tested the glycolytic phenotypes of tumor cells from PyMT and PyMT;*Zeb1*^*cKO*^ mice. Zeb1 depletion decreased the extracellular acidification rate (ECAR), an indicator of overall glycolytic flux, and enhanced the oxygen consumption rate (OCR), which reflects mitochondrial oxidative respiration (Fig. 2A and B). Moreover, Zeb1 depletion reduced the glucose uptake, pyruvate level, lactate production, and ATP level in PyMT;*Zeb1*^*cKO*^ cells compared to those in PyMT cells (Fig. 2C). Similar effects of Zeb1 on glycolytic activities were also confirmed in MDA-MB-231 (Fig. 2D–F, Fig. S3) and SUM-159 (Fig. S4) cells, which have been reported to exhibit the Warburg effect [4, 5].

### Zeb1 transcriptionally regulates glycolytic gene expression

**Fig. 3:**
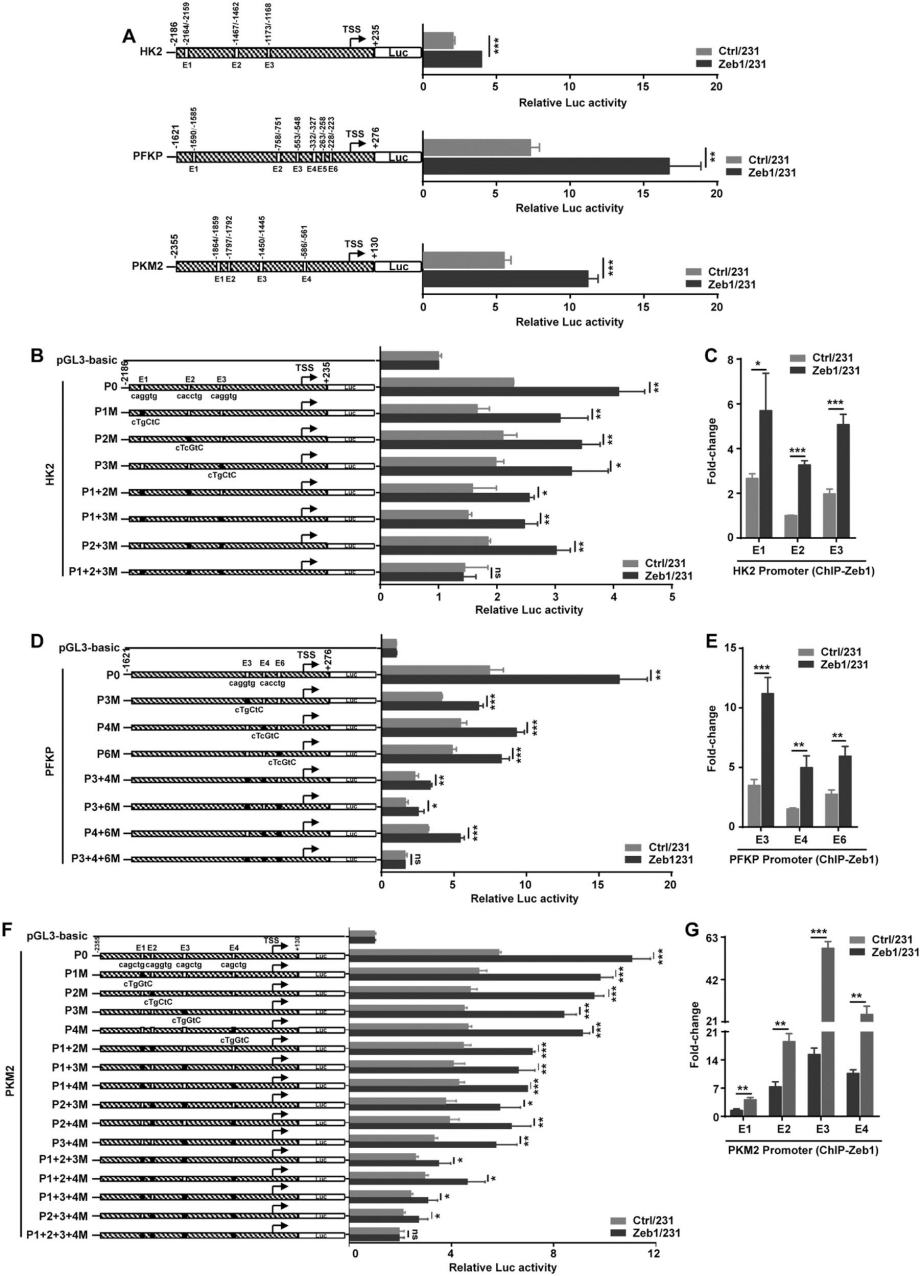
Zeb1 regulates glycolytic gene transcription. **A** Luciferase assay for the wild-type promoters of HK2 (−2186/+235), PKFP (−1621/+276) and PKM2 (−2355/+130) in Ctrl/231 and Zeb1/231 cells. **B** Luciferase assay for the E_2_-box-mutated promoters of HK2 in Ctrl/231 and Zeb1/231 cells. **C** ChIP assay for recruitment of Zeb1 to the endogenous HK2 promoter in Ctrl/231 and Zeb1/231 cells. **D** Luciferase assay for the E_2_-box-mutated promoters of PFKP in Ctrl/231 and Zeb1/231 cells. **E** ChIP assay for recruitment of Zeb1 to the endogenous PFKP promoter in Ctrl/231 and Zeb1/231 cells. **F** Luciferase assay for the E_2_-box-mutated promoters of PKM2 in Ctrl/231 and Zeb1/231 cells. **G** ChIP assay for recruitment of Zeb1 to the endogenous PKM2 promoter in Ctrl/231 and Zeb1/231 cells. Data are representative of three independent experiments and presented as mean ± SEM. *P < 0.05, **P < 0.01, ***P < 0.001 vs the respective control by an unpaired Student’s *t*-test.

To further test whether Zeb1 transcriptionally regulates the gene expression of HK2, PFKP, and PKM2, as shown in Fig. 3A, we constructed promoter reporters and searched up to ~2 kb of the promoter regions of these genes for putative Zeb1 binding sites [E_2_-boxes: CA(C/G)(C/G)TG]. The luciferase assay showed that Zeb1 overexpression increased the promoter activity of HK2, PFKP and PKM2 in MDA-MB-231 cells. Considering that the HK2, PFKP, and PKM2 promoters contained multiple Zeb1 binding sites, we performed ChIP to identify the putative response elements for endogenous Zeb1. The results indicated that endogenous Zeb1 was recruited to the binding sites E1, E2, and E3 in the HK2 promoter (Fig. S5A). Either E1, E2, or E3 was able to mediate Zeb1-induced transcriptional activation of the HK2 promoter, whereas simultaneous deletion or mutation of all Zeb1 binding sites completely eliminated this activity (Fig. 3B and Fig. S5B). Of note, overexpression of Zeb1 significantly increased its recruitment to E1, E2, and E3 elements in the HK2 promoter (Fig. 3C), demonstrating a predominant role for E_2_-boxes in the activation of HK2 transcription by Zeb1. Similarly, we further identified that E3, E4, and E6 sites in the PFKP promoter (Fig. S5C, D, Fig. 3D, E) and E1, E2, E3, and E4 sites in the PKM2 promoter (Fig. S5E, F, Fig. 3F, G) are crucial for Zeb1 transcriptional activation. These data collectively suggested that Zeb1 directly promotes glycolytic gene transcription by binding to the corresponding promoters in an E_2_ box-dependent manner.

### Zeb1 regulates aerobic glycolysis under hypoxia

**Fig. 4:**
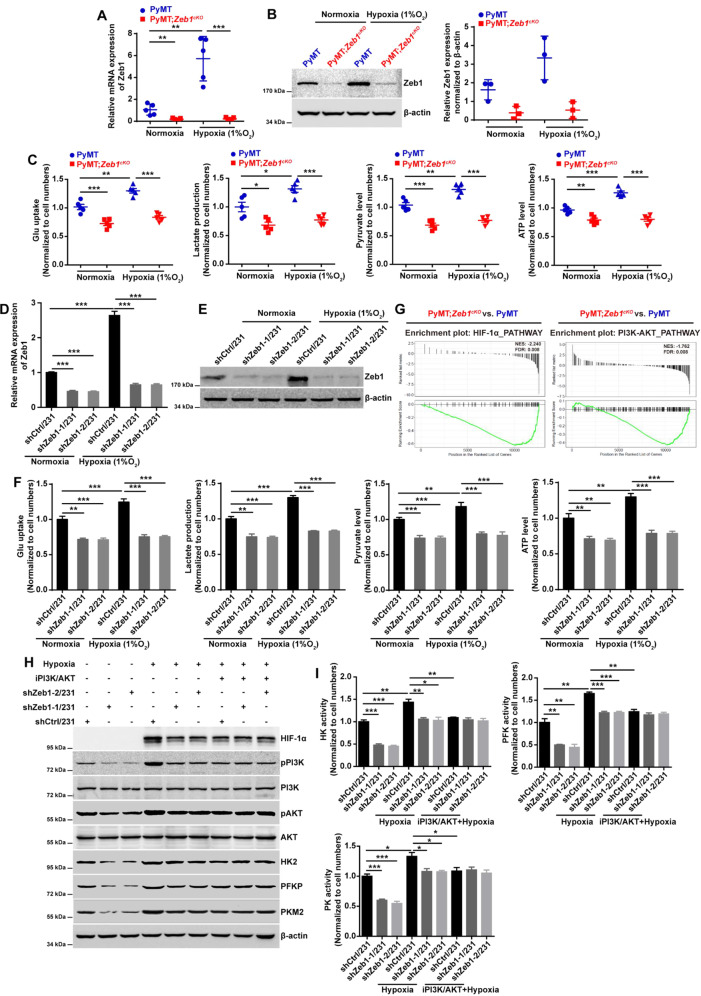
Zeb1 regulates aerobic glycolysis under hypoxia. **A**, **B** The relative (**A**) mRNA and (**B**) protein levels of Zeb1 in PyMT and PyMT;*Zeb1*^*cKO*^ cells under normoxic and hypoxic conditions. **C** The alternations in glucose uptake, pyruvate level, lactate production, and ATP level in PyMT and PyMT;*Zeb1*^*cKO*^ cells under normoxic and hypoxic conditions. **D**, **E** The relative (**D**) mRNA and (**E**) protein levels of Zeb1 in scramble shRNA-transfected (shCtrl/231) and Zeb1-specific shRNA-transfected (shZeb1/231) MDA-MB-231 cells under normoxic and hypoxic conditions. **F** The alternations in glucose uptake, pyruvate level, lactate production, and ATP level in shCtrl/231 and shZeb1/231 cells under normoxic and hypoxic conditions. **G** GSEA for transcriptome data from PyMT;*Zeb1*^*cKO*^ vs PyMT cells revealing enrichment of a gene signature associated with reduced HIF-1α and PI3K-Akt signaling pathway activities in PyMT;*Zeb1*^*cKO*^ breast cancer cells. NES, normalized enrichment score; FDR, false discovery rate. **H** The protein levels of HIF-1α, HK2, PFKP, and PKM2 in shCtr//231 and shZeb1/231 cells under normoxic and hypoxic conditions in response to a PI3K/Akt inhibitor LY294002. **I** The enzyme activities of HK, PFK, and PKM in shCtrl/231 and shZeb1/231 cells under normoxic and hypoxic conditions in response to LY294002. Dots depict individual samples in (**A**–**C**). Data are representative of five (**A**, **C**) or three (**B**, **D**–**F**, **H**, **I**) independent experiments and presented as mean ± SEM. *P < 0.05, **P < 0.01, ***P < 0.001 vs the respective control by an unpaired Student’s *t*-test.

Considering that hypoxia plays a pivotal role in ectopic Zeb1 expression in breast cancer [58], we determined whether Zeb1 regulates aerobic glycolysis under hypoxia. The results demonstrated that hypoxia stimulated Zeb1 expression at both the mRNA (Fig. 4A) and protein (Fig. 4B) levels in PyMT tumor cells, whereas this effect was strongly attenuated in PyMT;*Zeb1*^*cKO*^ cells. Notably, hypoxia-increased glycolytic phenotypes, including increases in the glucose uptake, pyruvate level, lactate production, and ATP level, were abolished in PyMT;*Zeb1*^*cKO*^ cells compared with PyMT tumor cells (Fig. 4C). Similar effects of Zeb1 on glycolytic activities under hypoxia were also observed in MDA-MB-231 (Fig. 4D–F) and SUM-159 (Fig. S6) cells with Zeb1 knockdown.

It has been shown that the PI3K/Akt signaling pathway can promote aerobic glycolysis by modulating the expression of HIF-1α [59], which is consistent with our GSEA analysis showing that *Zeb1* depletion was associated with decreased PI3K/Akt activity and a reduced HIF-1α signature in PyMT;*Zeb1*^*cKO*^ cells (Fig. 4G). Similarly, hypoxia led to upregulation of *p*-PI3K, *p*-Akt, and HIF-1α in control MDA-MB-231 cells, which was accompanied by increased expression and activity of HK2, PFKP, and PKM2 (Fig. 4H, I). However, these effects were significantly abolished in MDA-MB-231 cells with Zeb1 knockdown. Moreover, treatment with a PI3K/Akt inhibitor LY294002 blocked the hypoxia-induced upregulation of HIF-1α and activation of these glycolytic enzymes in control MDA-MB-231 cells, which were strongly attenuated by Zeb1 knockdown. Similar results were also obtained in SUM-159 cells (Fig. S7), collectively suggesting that Zeb1 exerts its biological effects to induce glycolytic activity under hypoxia via the PI3K/Akt/HIF-1α signaling axis.

**Fig. 5:**
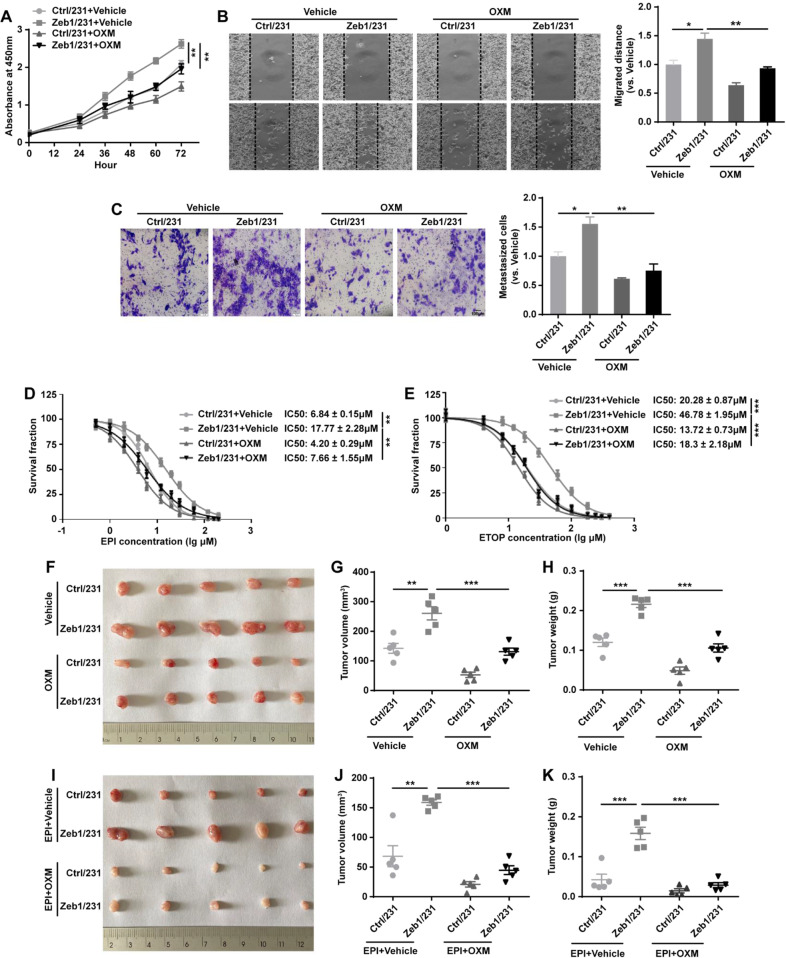
Zeb1-induced aerobic glycolysis contributes to breast cancer progression. **A** The cell viability of Ctrl/231 and Zeb1/231 cells by treatment with OXM. **B**, **C** The cell migration of Ctrl/231 and Zeb1/231 cells by treatment with OXM evaluated by (**B)** wound-healing and (**C)** transwell assays. **D**, **E** The cell viability of Ctrl/231 and Zeb1/231 cells by treatment with (**D)** EPI or (**E)** ETOP in the presence of OXM. **F** In vivo xenograft tumorigenicity of BALB/c mice injected with Ctrl/231 and Zeb1/231 cells by treatment with OXM. **G**, **H** Approximate tumor (**G**) volume and (**H**) weight of BALB/c mice injected with Ctrl/231 and Zeb1/231 cells by treatment with OXM. **I** In vivo xenograft tumorigenicity of BALB/c mice injected with Ctrl/231 and Zeb1/231 cells by treatment with EPI and/or OXM. **J**, **K** Approximate tumor (**J**) volume and (**K**) weight of BALB/c mice injected with Ctrl/231 and Zeb1/231 cells by treatment with EPI and/or OXM. Data are representative of five (**F**–**K**) or three (**A**–**E**) independent experiments and presented as mean ± SEM; **P < 0.01, ***P < 0.001 vs the respective control by one-way ANOVA followed by Tukey’s honestly significant difference test in (**A**) and (**D**, **E**). *P < 0.05, **P < 0.01, ***P < 0.001 vs the respective control by an unpaired Student’s *t*-test in (**B**, **C**, **G**, **H**, **J**, **K**).

### Zeb1-induced aerobic glycolysis contributes to breast cancer cell growth, metastasis, and chemoresistance

It has been established that dysfunctional aerobic glycolysis contributes to malignant tumor progression and therapeutic resistance [4, 5]. We thus investigated whether Zeb1-induced aerobic glycolysis affects breast cancer growth and metastasis. The results of CCK-8 (Fig. 5A), wound-healing (Fig. 5B) and transwell (Fig. 5C) assays indicated that ectopic Zeb1 expression promoted breast cancer cell viability and migration; however, treatment with the LDHA inhibitor OXM significantly weakened these effects. In addition, Zeb1-expressing MDA-MB-231 cells were relatively sensitive to the genotoxic agents epirubicin (EPI) and etoposide (ETOP) in the presence of OXM (Fig. 5D, E). We also performed these experiments in SUM-159 cells and obtained similar results (Fig. S8), collectively revealing that aerobic glycolysis contributes to tumor progression and chemoresistance in breast cancer cells with ectopic Zeb1 expression.

Next, to determine whether glycolysis plays a role in Zeb1-mediated regulation of tumor growth in vivo, we constructed a breast cancer xenograft model using female BALB/c nude mice by treatment with OXM and/or EPI. As expected, OXM treatment inhibited tumor growth in Ctrl/231 xenografts (Fig. 5F–H). Moreover, OXM strongly attenuated the ability of Zeb1 to promote tumor growth in Zeb1/231 xenografts, suggesting that glycolysis induced by Zeb1 is critical for breast cancer cell growth. Of note, the use of OXM and EPI in combination had greater effects to limit tumor volume and weight in Zeb1/231 xenografts (Fig. 5I–K). These observations together revealed that the inhibition of Zeb1-mediated aerobic glycolysis by OXM leads to chemosensitizing potency in vivo.

### Zeb1-induced aerobic glycolysis contributes to M2-like TAM polarization

**Fig. 6:**
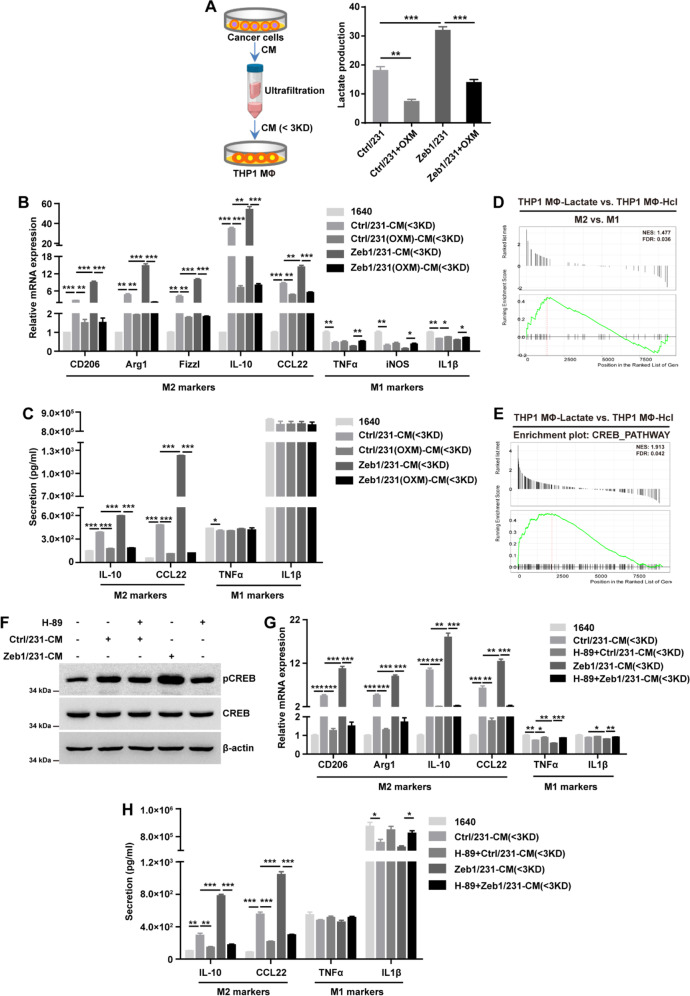
Zeb1-induced lactate production contributes to M2 TAM polarization. **A** Lactate concentration in fractionated CM from Ctrl/231 and Zeb1/231 cells treated with OXM. **B**, **C** The relative (**B**) mRNA and (**C**) protein levels of M1 and M2 macrophage markers in THP1 MΦ treated with fractionated CM from Ctrl/231 and Zeb1/231 cells in the presence of OXM. **D**, **E** GSEA of transcriptome data from THP1 MФ-lactate vs THP1 MФ-HCl cells revealing enrichment of a gene signature associated with increased (**D**) M2 TAM phenotypes and (**E**) CREB signaling pathway activity in THP1 MФ-lactate cells. NES, normalized enrichment score; FDR, false discovery rate. **F** The protein levels of phospho-CREB and total CREB in THP1 MΦ treated with fractionated CM from Ctrl/231 and Zeb1/231 cells in the presence of a PKA inhibitor H89. **G**, **H** The relative (**G)** mRNA and (**H)** protein levels of M1 and M2 macrophage markers in THP1 MΦ treated with fractionated CM from Ctrl/231 and Zeb1/231 cells in the presence of H89. Data are representative of three (**A**–**C**, **F**–**H**) independent experiments and presented as mean ± SEM. *P < 0.05, **P < 0.01, ***P < 0.001 vs the respective control by an unpaired Student’s *t*-test.

Considering that lactate is correlated with immune cell re-education in the TME [10], we aimed to validate whether cancer cell-derived lactate could modulate the polarization of TAMs. As shown in Fig. 6A, the <3-kDa fraction with lactate was obtained from conditioned medium of breast cancer cell cultures. We found that the lactate concentration was increased in CM from Zeb1-expressing MDA-MB-231 cells, which was significantly blocked by treatment with OXM (Fig. 6A). Of note, quantitative PCR analysis further demonstrated that the <3-kDa fraction derived from Zeb1-expressing MDA-MB-231 cells enhanced the expression of M2-like TAM markers including CD206, Arg1, Fizzl, IL-10, and CCL22 in THP-1 MΦ, and remarkably, this effect was abolished by OXM addition (Fig. 6B). However, alterations in M1 TAM markers such as TNF-α, iNOS, and IL-1β were not evident. These results were further confirmed by ELISA (Fig. 6C), showing that Zeb1-induced aerobic glycolysis contributes to M2-like TAM polarization via lactate production. We also performed these experiments in SUM-159 cells and obtained the same results (Fig. S9).

To further identify the potential mechanism involving in lactate-induced M2-like TAM polarization, we isolated total RNA from THP1 MΦ treated with lactate and performed RNA sequencing. GSEA results confirmed that lactate induction was associated with a significantly increased M2-like TAM signature compared with HCl control treatment (Fig. 6D). Of note, treatment of THP1 MΦ with lactate also enriched genes associated with the CREB-related signature (Fig. 6E). Considering that G protein-coupled receptor 132 (Gpr132) has been identified as a key macrophage sensor of lactate that mediates the interaction between breast cancer cells and TAMs [30], activation of the PKA/CREB signaling pathway might be involved in lactate-mediated M2-like TAM polarization. Indeed, treatment with H89 (a PKA inhibitor) blocked M2-like TAM phenotypes in THP1 MΦ cultured in CM from Zeb1-expressing MDA-MB-231 cells (Fig. 6F, G). These data thus revealed that breast cancer cells with ectopic Zeb1 expression produce lactate to induce M2-like TAM polarization via the PKA/CREB signaling axis.

### Zeb1 expression is positively correlated with aerobic glycolysis in human breast cancer

**Fig. 7:**
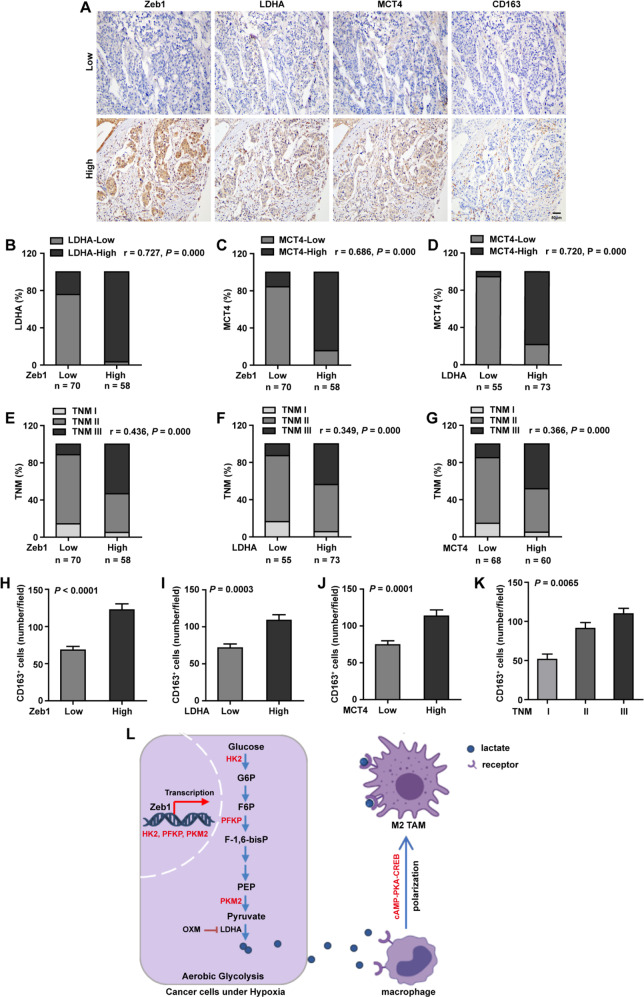
Zeb1 is positively correlated with aerobic glycolysis in human breast cancer. **A** Representative images of immunohistochemical staining for Zeb1, LDHA, MCT4, and CD163 in serial sections of the same tumor from two patients. Scale bars, 50 μm. **B** A positive correlation between the expression of Zeb1 and LDHA in 128 human breast cancer samples. *r* = 0.727, *P* = 0.000 by Spearman’s rank correction test. **C** A positive correlation between the expression of Zeb1 and MCT4 in human breast cancer samples. *r* = 0.686, *P* = 0.000 by Spearman’s rank correction test. **D** A positive correlation between the expression of LDHA and MCT4 in human breast cancer samples. *r* = 0.720, *P* = 0.000 by Spearman’s rank correction test. **E** A positive correlation between the expression of Zeb1 and TNM stages in 128 human breast cancer samples. *r* = 0.436, *P* = 0.000 by Spearman’s rank correction test. **F** A positive correlation between the expression of Zeb1 and TNM stages in human breast cancer samples. *r* = 0.349, *P* = 0.000 by Spearman’s rank correction test. **G** A positive correlation between the expression of LDHA and TNM stages in human breast cancer samples. *r* = 0.366, *P* = 0.000 by Spearman’s rank correction test. **H** A positive correlation between the expression of Zeb1 and CD163 in human breast cancer samples. *P* < 0.0001 by an unpaired Student’s *t*-test. **I** A positive correlation between the expression of LDHA and CD163 in human breast cancer samples. *P* = 0.0003 by an unpaired Student’s *t*-test. **J** A positive correlation between the expression of MCT4 and CD163 in human breast cancer samples. *P* = 0.0001 by an unpaired Student’s *t*-test. **K** A positive correlation between the expression of CD163 and TNM stages in human breast cancer samples. *P* = 0.0065 by one-way analysis of variance. **L** A working model illustrating that Zeb1-induced metabolic reprogramming of glycolysis regulates M2 TAM polarization in breast cancer.

To further strengthen the pathological correlation between Zeb1 expression and aerobic glycolysis in human breast cancer, we performed immunohistochemical staining for Zeb1, LDHA and MCT4 (a lactate transporter marker) in 128 samples of primary breast carcinoma (Fig. 7A). The results indicated a strong positive correlation between the expression of Zeb1 and LDHA (Fig. 7B). We also observed increased expression of Zeb1 and LDHA in tumors with high MCT4 activity (Fig. 7C, D). Importantly, we demonstrated concomitantly high expression of Zeb1, LDHA, and MCT4 in breast cancer patients with an advanced TNM stage (Fig. 7E–G), highlighting that Zeb1-mediated dysregulation of aerobic glycolysis is functionally linked to breast tumor malignancy.

In addition, to examine the accumulation and polarization of M2-like TAMs in tumors, the expression of CD163 (a M2-like TAM marker) was detected by immunohistochemical staining. Our data revealed strong correlations between elevated expression of Zeb1, LDHA or MCT4 and high levels of CD163 (Fig. 7H–J). Moreover, tumors in an advanced TNM stage had increased levels of CD163^+^ M2-like TAM in the TME (Fig. 7K). Taken together, these results are consistent with previous findings showing that highly malignant tumors are often correlated with increased aerobic glycolysis and enriched with abundant M2-like TAMs [60].

## Discussion

The metabolic reprogramming of aerobic glycolysis gives cancer cells a growth advantage by providing energy for cancer cell growth. Therefore, the identification of oncogenic signaling pathways responsible for the reprogramming of glucose metabolism may be translated into improved antineoplastic therapies. Based on our findings, we proposed that Zeb1 directly promotes the expression of key glycolytic genes that facilitate the Warburg effect and aggressiveness of breast cancer in vitro and in vivo. Mechanistically, we showed that Zeb1 regulates glucose uptake and the level of lactate, which is a metabolite that enhances tumor growth and induces an immunosuppressive TME. Therefore, our data uncover an alternative mechanism of Zeb1-mediated aerobic glycolysis as a driver of breast tumorigenesis by stimulating tumor–macrophage interplay (Fig. 7L).

Zeb1 promotes cancer progression through a combination of genetic, epigenetic, and transcriptional mechanisms [42, 46]. Our data on Zeb1-dependent gene expression signatures further revealed that the regulatory potential of Zeb1 is not limited to effects on a few crucial downstream target genes but rather leads to global reprogramming of gene expression patterns and controls not only EMT but also other programs and pathways, such as aerobic glycolysis. In particular, our results demonstrated that Zeb1, as a transcription factor, directly increases the expression of multiple glycolytic genes, thus promoting the Warburg effect and tumor aggressiveness in vitro and in vivo. These observations fit with the reduced cellular plasticity and decreased tumorigenic capacity of PyMT;*Zeb1*^*cKO*^ tumors compared to those of PyMT tumors [50]. Since Zeb1 regulates expression of the glycolytic rate-determining enzymes HK2, PFKP and PKM2 that modulate cancer cell proliferation and/or apoptosis [4–7], the function of Zeb1 in aerobic glycolysis at least partly explains the phenotypes induced by Zeb1 depletion in breast cancer cells. Moreover, Zeb1-linked plasticity is verified by its impact on additional metabolic pathways. First, in pancreatic cancer, plasticity in switching between basic energy pathways is strongly compromised in Zeb1-depleted cancer cells, which display both decreased oxidative phosphorylation (OXPHOS) and a reduced glycolytic reserve; this plasticity might be critical for the colonization step [40]. This is consistent with our conclusion that cancer cells might use aerobic glycolysis more than oxidative respiration to meet their energy requirements. Second, Zeb1 also provides a bridge between lipid peroxide vulnerability and a high-mesenchymal cell state associated with resistance to multiple treatment modalities by modulating master regulators of lipid biology, including peroxisome proliferator-activated receptor-γ (PPARγ) and phospholipid glutathione peroxidase (GPX4) [41]. Taken together with our present study, these findings shed new light on the mechanisms of Zeb1 in cancer metabolism regulation.

Hypoxia represents a key microenvironmental stressor that governs multiple stronger immunosuppressive phenomena associated with tumor progression [27, 60]. A growing body of evidence suggests that protumoral M2-like TAMs preferentially accumulate in the hypoxic areas of the TME, and this process plays a prominent role in tumorigenesis. However, precisely how M2-like TAMs are educated by the hypoxic TME remains elusive. In this study, we proposed a mechanism by which hypoxia-induced Zeb1 potentiates the protumoral function of M2-like TAMs through promotion of lactate secretion to drive the formation of a tumor-supportive TME. This is consistent with a previous investigation showing that M2-like TAMs in the stroma always surround Zeb1-positive cells in ovarian and cervical cancers [61, 62], supporting the potent roles of Zeb1 in the recruitment and polarization of M2-like TAMs. On the other hand, our RNA-sequencing results using THP1 MΦ indicated that several Zeb1-inducing factors, including VEGFA [50], EGF [63], and CCL18 [64], were strongly upregulated in response to lactate (data not shown). These observations provided evidence that hypoxia-induced Zeb1 modulates the interaction between breast cancer cells and M2-like TAMs and that this crosstalk, in turn, regulates Zeb1 expression itself. Our study thus revealed a possible mechanism of M2-like TAM infiltration into the hypoxic TME via Zeb1-driven aerobic glycolysis in cancer cells. The unexpected role of hypoxia-induced Zeb1 expression established by our study might offer additional approaches for targeting immunosuppressive TAMs in breast cancer.

Recent studies have shown that reacidification with lactate but not HCl in OXM-pretreated breast cancer cells rescues the phenotypes of M2-like TAM polarization, highlighting that lactate, not simply a pH drop, triggers the effects on macrophages [65]. In agreement, our GSEA results confirmed that treatment with lactate rather than HCl was associated with an increased M2-like TAM signature in THP1 MΦ. Moreover, Chen et al. previously reported that Gpr132, a member of the pH-sensing G protein-coupled receptor family, exerts a key function in M2-like TAM polarization during breast cancer metastasis. This Gpr132-dependent activity also resides in the <3-kDa fraction of cancer cell CM and is largely attributed to lactate [30]. Together with our GSEA data showing that lactate induction was specifically enriched among genes associated with the CREB-related signature, these findings highlighted PKA/CREB signaling as a lactate-targeting pathway in macrophages. Indeed, inhibition of the PKA activity resulted in significant blockade of M2-like TAM phenotypes induced by lactate in THP1 MΦ. Our conclusion is also consistent with previous studies showing that activation of the PKA/CREB signaling pathway contributes to macrophage polarization and remodeling of the immunosuppressive TME during cancer progression [66, 67]. Our work might open an exciting path to future investigations on the functional roles of the lactate-PKA/CREB axis in the crosstalk between metabolism and immunity.

In summary, our findings demonstrated that breast cancer cells exhibit aberrant glycolytic metabolism mediated through a Zeb1-dependent mechanism that confers aggressive and immunosuppressive properties to tumors. Importantly, our study introduced the potential therapeutic approaches that disrupt the link between Zeb1-induced aerobic glycolysis and carcinogenesis, eventually leading to improvement of the clinical outcomes of patients with aggressive breast cancer.

## Supplementary information

'marked-up’version of the revised manuscript

supplementary information

Reproducibility Checklist

## Data Availability

RNA-seq data have been deposited at GEO DataSets (GSE189779). Previously published RNA-seq datasets can be found under the accession number PRJNA511636. Additional data are available from the corresponding author upon reasonable request.
