# Supplementary material for: Zeb1-induced metabolic reprogramming of glycolysis is essential for macrophage polarization in breast cancer
Source: Cell Death Dis. 2022 Mar 4;13(3):206. doi: 10.1038/s41419-022-04632-z (PMC8897397; doi:10.1038/s41419-022-04632-z)
Supplement: Supplementary file 2 — supplementary information [file 41419_2022_4632_MOESM2_ESM.docx]

**Supplementary Information**

**Zeb1-induced metabolic reprogramming of glycolysis is essential for macrophage polarization in breast cancer**

Content:

1. Supplementary Tables

2. Supplementary Figures and Figure Legends

**Supplementary Table 1. Sequences of the oligonucleotides for shRNA, mice genotyping, real-time PCR, and site-mutagenesis (5’-3’).**

| Construction of shRNA vector | |
| --- | --- |
| shZeb1-1 | CGGCGCAATAACGTTACAAAT |
| shZeb1-2 | GGCGCAATAACGTTACAAA |
| Construction of promoter | |
| HK2-P-0.7K-F | CCAAGCTTGGGAGTGAGGTTAGCCAGA |
| HK2-P-1.4K-F | GGGGTACCCGTGATCCGTCCACCTCA |
| HK2-P-1.9K-F | GGGGTACCCTCAACAACCCGTTACCAAC |
| HK2-P-2.4K-F | GGGGTACCCAGTTATGAGGCTGTTCCAGT |
| HK2-P-R | CCAAGCTTCAGATTTCAAGAGACATGACATTA |
| PFKP-P-0.5K-F | GGGGTACCTGGCGCTACCCCTGCCTC |
| PFKP-P-0.6K-F | GGGGTACCACCAGAGTCCTCCAGCCCA |
| PFKP-P-0.7K-F | GGGGTACCCCCGCAAACGATGTGACC |
| PFKP-P-0.9K-F | GGGGTACCAGGAATCTTGCGGGCCACT |
| PFKP-P-1.3K-F | GGGGTACCGAGGTCAGGCAGAGCGTACAG |
| PFKP-P-1.9K-F | GGGGTACCTTCAGTGGTTTAGCGGGAGTG |
| PFKP-P-R | GAAGATCTATGCCCTGGTAGCCCTCGT |
| PKM2-P-0.2K-F | GGGGTACCTGGGTCAAGGGCTGTTTG |
| PKM2-P-1.2K-F | GGGGTACCCCTGTTCCGTGCTCGTTT |
| PKM2-P-1.7K-F | GGGGTACCCCTCCTGAAGGTGACTGC |
| PKM2-P-2.5K-F | GGGGTACCCACGACAAAGGTGAAAACAGA |
| PKM2-P-R | GAAGATCTCATCTTCTAATCCAGTGTGCTT |
| Site-Directed Mutagenesis of promoter | |
| HK2-Mut 1-F | GGGGTACCCAGTTATGAGGCTGTTCCAGTCTGCTCAGAGATAACTGGGCA |
| HK2-Mut 1-R | CCAAGCTTCAGATTTCAAGAGACATGACATTA |
| HK2-Mut 2-F | AATGCCTCGTCCTTGCCCTTGAAT |
| HK2-Mut 2-R | GCAAGGACGAGGCATTAGACAGCA |
| HK2-Mut 3-F | GATTACTGCTCTGAGCCACCGCGC |
| HK2-Mut 3-R | GCTCAGAGCAGTAATCCCAGCACT |
| PFKP-Mut 3-F | GCGTCCTGCTGGGGTCTCCGCGCC |
| PFKP-Mut 3-R | GACCCCAGCAGGACGCGGAGGCTC |
| PFKP-Mut 4-F | TGCCCCTCGTCTGGCGCTCCCGTC |
| PFKP-Mut 4-R | CGCCAGACGAGGGGCAGGGGCAGC |
| PFKP-Mut 6-F | CCGCCCTCGTCTGGCGCTACCCCT |
| PFKP-Mut 6-R | CGCCAGACGAGGGCGGGTCCCTGC |
| PKM2-Mut 1-F | CGGGACTGGTCGGGTGGCCTGGGC |
| PKM2-Mut 1-R | CACCCGACCAGTCCCGGCCTACCT |
| PKM2-Mut 2-F | GGCAACTGCTCGCCCGGCGCCCGG |
| PKM2-Mut 2-R | CGGGCGAGCAGTTGCCGCCAAGTT |
| PKM2-Mut 3-F | CACAGCTGGTCCACGCGCCGTGGC |
| PKM2-Mut 3-R | GCGTGGACCAGCTGTGCAAGGAGC |
| PKM2-Mut 4-F | GGGGGCTGGTCGAGACTTACGTAA |
| PKM2-Mut 4-R | GTCTCGACCAGCCCCCGGGCCGCC |
| Mice genotyping | |
| PyMT-F | GGAAGCAAGTACTTCACAAGGG- |
| PyMT-R | GGAAAGTCACTAGGAGCAGGG |
| Cre-F | ATTTGCCTGCATTACCGGTC |
| Cre-R | ATCAACGTTTTCTTTTCG G |
| Zeb1-LoxP-F | GTCTATCCAGAATCTTCCCATGAC |
| Zeb1-LoxP-R | CTGTCAATCTCTGGCCTCTACATG |
| Quantitative PCR | |
| ACTIN-F | CATGTACGTTGCTATCCAGGC |
| ACTIN-R | CTCCTTAATGTCACGCACGAT |
| ZEB1-F | CAGCTTGATACCTGTGAATGGG |
| ZEB1-R | TATCTGTGGTCGTGTGGGACT |
| PFKP-F | GCATGGGTATCTACGTGGGG |
| PFKP-R | CTCTGCGATGTTTGAGCCTC |
| HK2-F | TTGACCAGGAGATTGACATGGG |
| HK2-R | CAACCGCATCAGGACCTCA |
| PKM2-F | ATAACGCCTACATGGAAAAGTGT |
| PKM2-R | TAAGCCCATCATCCACGTAGA |
| mACTIN-F | TGGCATTGTTACCAACTGGGAC |
| mACTIN-R | GAAGGTCTCAAACATGATCTGG |
| mZEB1-F | GCTGGCAAGACAACGTGAAAG |
| mZEB1-R | GCCTCAGGATAAATGACGGC |
| mPFKP-F | TGGAAGCCAAATGGGACT |
| mPFKP-R | CACCACGTTGAGGTAGGAAT |
| mHK2-F | TTTAGGTCAGTCGGCGTTTC |
| mHK2-R | CGGAGGTGGGATCTGCTAG |
| mPKM2-F | GAAGGCGTCCGCAGGTTT |
| mPKM2-R | GAATCTCAATGCCCAGGTCAC |
| CD206-F | GGGTTGCTATCACTCTCTATGC |
| CD206-R | TTTCTTGTCTGTTGCCGTAGTT |
| Arg1-F | ACCATAGGGATTATTGGAGC |
| Arg1-R | TGTCATTAGGGATGTCAGCA |
| Fizzl-F | AGAGTACAGTCCCTCTCC |
| Fizzl-R | AACCACAGCCATAGCCACAA |
| IL-10-F | ATGCACAGCTCAGCACTGC |
| IL-10-R | CTTGATGTCTGGGTCTTGGTT |
| CCL22-F | AGCCAATGAAGAGCCTAC |
| CCL22-R | GCAGAGGATGGGTTAGAG |
| TNFα-F | CGAGTGACAAGCCTGTAGCC |
| TNFα-R | TGAAGAGGACCTGGGAGTAGAT |
| iNOS-F | TTCAGTATCACAACCTCAGCAAG |
| iNOS-R | TGGACCTGCAAGTTAAAATCCC |
| IL1β-F | GCTTATTACAGTGGCAATGAGGAT |
| IL1β-R | CCTCGTTATCCCATGTGTCG |
| Quantitative ChIP | |
| HK2-E1-F | CAGTTATGAGGCTGTTCCAGT |
| HK2-E1-R | TAAAATGGTGCCTGACGC |
| HK2-E2-F | ACTCAACAACCCGTTACC |
| HK2-E2-R | GAAAGAGACTCCATTTCA |
| HK2-E3-F | ACCTCGTGATCCGTCCAC |
| HK2-E3-R | TGACGTTCCACTCAGATACCT |
| PFKP-E3-F | GGAATCTTGCGGGCCACT |
| PFKP-E3-R | GGGCGGGATAGAACGCC |
| PFKP-E4-F | TGCTCCCGGCGTTCTATC |
| PFKP-E4-R | GGCTGGAGGACTCTGGTTGG |
| PFKP-E6-F | CGCTCCCGTCATCTCTA |
| PFKP-E6-R | GCCAATGGGCGGTGACG |
| PKM2-E1-F | ACTTCGCAGTCCCTAGTT |
| PKM2-E1-R | GGTGTCTTTTCTTGGCT |
| PKM2-E2-F | CCCCGGAACCCATAAATC |
| PKM2-E2-R | TAAGAAACCTGATGACCAATGG |
| PKM2-E3-F | CCTCCTGAAGGTGACTG |
| PKM2-E3-R | TACGCTGCAAAGACGAAG |
| PKM2-E4-F | CCCCCCTTTTCCATCACC |
| PKM2-E4-R | GCAGGCCGCCCATCTAA |

**Supplementary Table 2. List of antibodies with their sources and experimental conditions.**

| Marker | Species | Application | Manufacturer | Catalog No. | Dilution |
| --- | --- | --- | --- | --- | --- |
| anti-ZEB1 | Rabbit | IHC | Abcam | ab87280 | 1:100 |
|  |  | IB/ChIP | proteintech | 21544-1-AP | 1:1000/1μg |
| anti-HK2 | Rabbit | WB | CST | 2867 | 1:1000 |
| anti-PFKP | Rabbit | WB | Proteintech | 13389-1-AP | 1:1000 |
| anti-PKM2 | Rabbit | WB | CST | 4053 | 1:1000 |
| anti-pPI3K | Rabbit | WB | Affinity | AF3241 | 1:1000 |
| anti-PI3K | Rabbit | WB | Abcam | Ab191606 | 1:1000 |
| anti-pAKT | Rabbit | WB | CST | #4060 | 1:1000 |
| anti-AKT | Rabbit | WB | CST | #4685 | 1:1000 |
| anti-pCREB | Abcam | WB | Abcam | Ab32096 | 1:1000 |
| anti-CREB | Rabbit | WB | CST | #9197 | 1:1000 |
| anti-HIF1α | Rabbit | WB | CST | #36169 | 1:1000 |
| anti-MCT4 | Rabbit | IHC | Proteintech | 22787-1-AP | 1:100 |
| anti-LDHA | Rabbit | IHC | Proteintech | 19987-1-AP | 1:100 |
| anti-CD163 | Rabbit | IHC | Abcam | Ab182422 | 1:200 |
| anti-β-actin | Mouse | IB | Santa Cruz | sc-47778 | 1:1000 |



**Supplementary Figure 1. Zeb1 depletion decreases the expression of glycolytic gene.** (**A** and **B**) The relative (**A**) mRNA and (**B**) protein levels of Zeb1, HK2, PFKP and PKM2 in scramble shRNA-transfected (shCtrl/231) and Zeb1-specific shRNA-transfected (shZeb1/231) MDA-MB-231 cells. (**C**) The enzyme activities of HK, PFK and PKM in shCtrl/231 and shZeb1/231 cells. Data are representative of three independent experiments and presented as mean ± SEM; **P* < 0.05, ***P* < 0.01, ****P* < 0.001 vs the respective control by an unpaired Student’s *t*-test.





**Supplementary Figure 2. Zeb1 is a key regulator of glycolytic gene expression.** (**A** and **B**) The relative (**A**) mRNA and (**B**) protein levels of HK2, PFKP and PKM2 in empty vector-expressing (Ctrl/159) and Zeb1-expressing (Zeb1/159) SUM-159 cells. (**C**) The enzyme activities of HK, PFK and PKM in Ctrl/159 and Zeb1/159 cells. (**D** and **E**) The relative (**D**) mRNA and (**E**) protein levels of Zeb1, HK2, PFKP and PKM2 in Zeb1-interfered SUM-159 cells. (**F**) The enzyme activities of HK, PFK and PKM in scramble shRNA-transfected (shCtrl/159) and Zeb1-specific shRNA-transfected (shZeb1/159) SUM-159 cells. Data are representative of three independent experiments and presented as mean ± SEM; **P* < 0.05, ***P* < 0.01, ****P* < 0.001 vs the respective control by an unpaired Student’s *t*-test.



**Supplementary Figure 3. Zeb1 depletion inhibits aerobic glycolysis.** (**A** and **B**) The alternations in (**A**) ECAR and (**B**) OCR in shCtrl/231 and shZeb1/231 cells. (**C**) The alternations in glucose uptake, pyruvate level, lactate production, and ATP level in shCtrl/231 and shZeb1/231 cells. Data are representative three independent experiments and presented as mean ± SEM; ***P* < 0.01, ****P* < 0.001 vs the respective control by an unpaired Student’s *t*-test.





**Supplementary Figure 4. Zeb1 regulates aerobic glycolysis.** (**A** and **B**) The alternations in (**A**) ECAR and (**B**) OCR in Ctrl/159 and Zeb1/159 cells. (**C**) The alternations in glucose uptake, pyruvate level, lactate production, and ATP level in Ctrl/159 and Zeb1/159 cells. (**D** and **E**) The alternations in (**D**) ECAR and (**E**) OCR in shCtrl/159 and shZeb1/159 cells. (**F**) The alternations in glucose uptake, pyruvate level, lactate production, and ATP level in shCtrl/159 and shZeb1/159 cells. Data are representative of three independent experiments and presented as mean ± SEM; **P* < 0.05, ***P* < 0.01, ****P* < 0.001 vs the respective control by an unpaired Student’s *t*-test.





**Supplementary Figure 5. Zeb1 regulates glycolytic gene transcription.** (**A**) ChIP assay for Zeb1 recruitment to the endogenous HK2 promoter in MDA-MB-231 cells. (**B**) Luciferase assay for the wild-type and E_2_-box-deleted promoters of HK2 in Ctrl/231 and Zeb1/231 cells. (**C**) ChIP assay for Zeb1 recruitment to the endogenous PFKP promoter in MDA-MB-231 cells. (**D**) Luciferase assay for the wild-type and E_2_-box-deleted promoters of PFKP in Ctrl/231 and Zeb1/231 cells. (**E**) ChIP assay for Zeb1 recruitment to the endogenous PKM2 promoter in MDA-MB-231 cells. (**F**) Luciferase assay for the wild-type and E_2_-box-deleted promoters of PKM2 in Ctrl/231 and Zeb1/231 cells. Data are representative of three independent experiments and presented as mean ± SEM; **P* < 0.05, ***P* < 0.01, ****P* < 0.001 vs the respective control by an unpaired Student’s *t*-test.





**Supplementary Figure 6. Zeb1 regulates aerobic glycolysis under hypoxia.** (**A** and **B**) The relative (**A**) mRNA and (**B**) protein levels of Zeb1 in shCtrl/159 and shZeb1/159 cells under normoxic and hypoxic conditions. (**C**) The alternations in glucose uptake, pyruvate level, lactate production, and ATP level in shCtrl/159 and shZeb1/159 cells under normoxic and hypoxic conditions. Data are representative of three independent experiments and presented as mean ± SEM; **P* < 0.05, ***P* < 0.01, ****P* < 0.001 vs the respective control by an unpaired Student’s *t*-test.





**Supplementary Figure 7. Zeb1 regulates aerobic glycolysis under hypoxia via PI3K/AKT pathway.** (**A**) The protein levels of HIF-1α, HK2, PFKP, and PKM2 in shCtrl/159 and shZeb1/159 cells under normoxic and hypoxic conditions in response to a PI3K/Akt inhibitor LY294002. (**B**) The enzyme activities of HK, PFK and PKM in shCtrl/159 and shZeb1/159 cells under normoxic and hypoxic conditions in response to LY294002. Data are representative of three independent experiments and presented as mean ± SEM; **P* < 0.05, ***P* < 0.01 vs the respective control by an unpaired Student’s *t*-test.


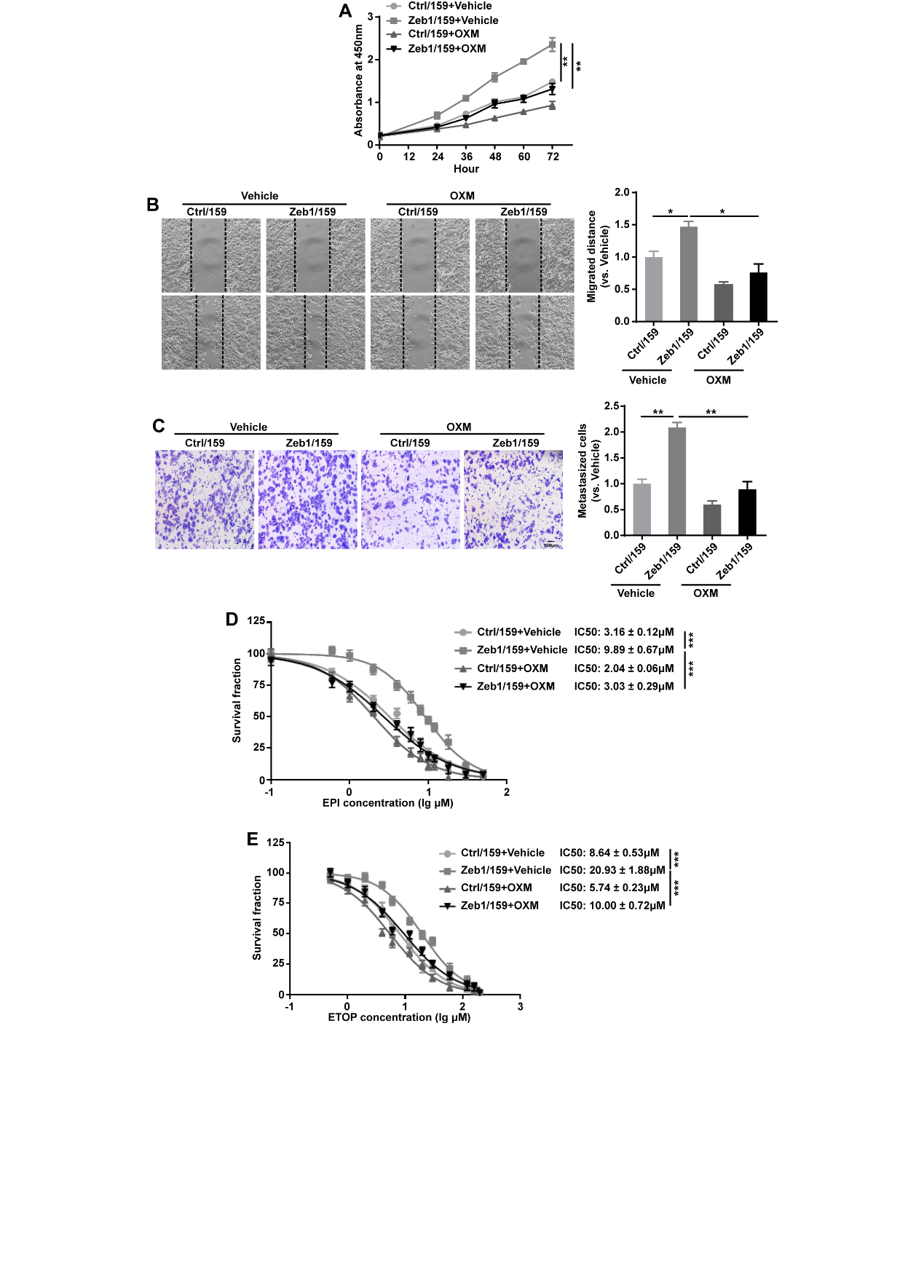


**Supplementary Figure 8. Zeb1-induced aerobic glycolysis contributes to breast cancer progression.** (**A**) The cell viability of Ctrl/159 and Zeb1/159 cells by treatment with OXM. (**B** and **C**) The cell migration of Ctrl/159 and Zeb1/159 cells by treatment with OXM evaluated by (**B**) wound-healing and (**C**) transwell assays. (**D** and **E**) The cell viability of Ctrl/159 and Zeb1/159 cells by treatment with (**D**) EPI or (**E**) ETOP in the presence of OXM. Data are representative of three independent experiments and presented as mean ± SEM; ***P* < 0.01, ****P* < 0.001 vs the respective control by one-way ANOVA followed by Tukey’s honestly significant difference test in A and D-E. **P* < 0.05, ***P* < 0.01 vs the respective control by an unpaired Student’s *t*-test in B and C.





**Supplementary Figure 9. Zeb1-induced lactate production contributes to M2 TAM polarization.** (**A**) Lactate concentration in fractionated CM from Ctrl/159 and Zeb1/159 cells by treatment with OXM. (**B** and **C**) The relative (**B**) mRNA and (**C**) protein levels of M1 and M2 macrophage markers in THP1 Φ by treatment with fractionated CM from Ctrl/159 and Zeb1/159 cells in the presence of OXM. (**D**) The relative protein levels of M1 and M2 macrophage markers in THP1 Φ by treatment with fractionated CM from Ctrl/159 and Zeb1/159 cells in the presence H89. Data are representative of three independent experiments and presented as mean ± SEM; **P* < 0.05, ***P* < 0.01, ****P* < 0.001 vs the respective control by an unpaired Student’s *t*-test.
